# Icaritin enhances mESC self-renewal through upregulating core pluripotency transcription factors mediated by ERα

**DOI:** 10.1038/srep40894

**Published:** 2017-01-16

**Authors:** Wing Pui Tsang, Fengjie Zhang, Qiling He, Waijiao Cai, Jianhua Huang, Wai Yee Chan, Ziyin Shen, Chao Wan

**Affiliations:** 1 Key Laboratory for Regenerative Medicine, Ministry of Education, School of Biomedical Sciences, Faculty of Medicine, The Chinese University of Hong Kong, Hong Kong SAR, China; 2 School of Biomedical Sciences Core Laboratory, Institute of Stem Cell, Genomics and Translational Research, Shenzhen Research Institute, The Chinese University of Hong Kong, Shenzhen 518057, China; 3 Department of Microbiology, University of Alabama at Birmingham, Birmingham, AL 35294, USA; 4 Institute of Integrative Chinese and Western Medicine, Huashan Hospital, Fudan University, Shanghai 200040, China

## Abstract

Utilization of small molecules in modulation of stem cell self-renewal is a promising approach to expand stem cells for regenerative therapy. Here, we identify Icaritin, a phytoestrogen molecule enhances self-renewal of mouse embryonic stem cells (mESCs). Icaritin increases mESCs proliferation while maintains their self-renewal capacity *in vitro* and pluripotency *in vivo*. This coincides with upregulation of key pluripotency transcription factors OCT4, NANOG, KLF4 and SOX2. The enhancement of mESCs self-renewal is characterized by increased population in S-phase of cell cycle, elevation of Cylin E and Cyclin-dependent kinase 2 (CDK2) and downregulation of p21, p27 and p57. PCR array screening reveals that caudal-related homeobox 2 (Cdx2) and Rbl2/p130 are remarkably suppressed in mESCs treated with Icaritin. siRNA knockdown of Cdx2 or Rbl2/p130 upregulates the expression of Cyclin E, OCT4 and SOX2, and subsequently increases cell proliferation and colony forming efficiency of mESCs. We then demonstrate that Icaritin co-localizes with estrogen receptor alpha (ERα) and activates its nuclear translocation in mESCs. The promotive effect of Icaritin on cell cycle and pluripotency regulators are eliminated by siRNA knockdown of ERα in mESCs. The results suggest that Icaritin enhances mESCs self-renewal by regulating cell cycle machinery and core pluripotency transcription factors mediated by ERα.

Embryonic stem cells (ESCs) are pluripotent stem cells originated from the inner cell mass of blastocyst, an early-stage preimplantation embryo^1^,^2^. ESCs are characterized by the presence of core pluripotency transcription factors including OCT4^3^, NANOG^4^,^5^,^6^, SOX2^7^ and KLF4^8^,^9^. These factors have been developed as powerful tools for generation of induced pluripotent stem cells (iPSCs) from mouse or human fibroblasts^8^. Under defined conditions, ESCs differentiate into all the lineages of the three germ layers: ectoderm, endoderm and mesoderm. ESCs have been served as an excellent platform for the investigation of embryonic development, disease modeling, drug screen and regenerative therapy. Clinical trials have been approved for using human ESCs (hESCs) for the treatment of difficult diseases such as spinal cord injury^10^,^11^.

The application of ESCs relies on the efficient expansion of the cells and elucidation of molecular mechanisms controlling their self-renewal and differentiation. ESCs are typically maintained in culture with mouse embryonic fibroblasts (MEFs) as feeder cells and/or exogenous factors. A compelling body of studies has been performed to identify cytokine/receptor signaling pathways controlling ESCs self-renewal. Among which the leukemia inhibitory factor (LIF)-gp130 receptor pathway through activating signal transducer and activator of transcription 3 (STAT3)^12^ has been identified as an essential self-renewal pathway for mESCs, while the bone morphogenetic proteins (BMPs)-Smad pathway was documented to exert combinatorial activities on mESCs in presence of LIF to suppress lineage differentiation through activation of the transcription factor Id (inhibitor-of-differentiation)^13^.

Using genetic manipulation or chemical screen approaches, multiple pluripotency regulators or small molecules have been defined to promote long-term maintenance of ESCs. For example, an inducible transgene containing key pluripotency transcription factors Oct4, Sox2, Klf4 and c-myc has been shown to promote the expansion of ESCs^14^. This kind of genetic manipulation needs a strict fine tuning of gene expression or the cells are subjected to undesired lineage differentiation. For example, a small increase in OCT4 protein level (i.e. 2-fold) induces ESC to differentiate into cells that express endoderm and mesoderm markers. In contrast, decreasing OCT4 expression leads to trophectoderm-like cells formation^15^. Similarly, solely alternation of other key transcription factors like SOX2, KLF4 or NANOG dramatically affects the fate of ESCs differentiation^16^. Apart from genetic manipulation, chemical approaches, in particular, the application of small molecules, offers new perspective for effective ESCs expansion and targeted differentiation for regenerative therapeutics. It is believed that such small molecules, no matter obtained from natural products or chemically synthesized, have advantages over the genetic approach such as more convenient to use, adjustable biological activities by optimized concentrations and combinations of multiple drugs^17^,^18^. It is well defined that combination of two small molecule inhibitors (2i) for mitogen-activated protein kinase (MAPK) and glycogen synthase kinase-3 (GSK3) with LIF (2i/LIF) provides an optimal environment for the long-term self-renewal of mESCs^19^,^20^ and promotes generation of iPSCs from partially reprogrammed cells (pre-iPSCs)^21^. A novel synthetic molecule pluripotin, is shown to maintain long-term self-renewal of mESCs in the absence of feeder cells through dual inhibition of differentiation-inducing proteins Ras GTPase-activating protein and ERKs^17^. These findings provide new insights into environmental control of ESCs biological behavior and may help to define new culture conditions free from feeders, serum and cytokines, and thus facilitate the potential application of ESCs in drug screen and regenerative medicine. However, investigations on small molecules from natural products such as phytomolecules or active ingredients of herbal medicines that regulate the self-renewal of ESCs remain lacking.

In this study, using phenotypic cellular screen assays, we identify that Icaritin, a phytoestrogen molecule from *Herba Epimedii*, promotes the self-renewal of mESCs. This molecule appears to act through inhibition of CDX2 and p130 dependent pathways which in turn activates the Cyclin E/CDK2 signaling and core pluripotency transcription factors, subsequently facilitates cell cycle G1/S phase transition and self-renewal of mESCs. At molecular level, Icaritin interacts with ERα and activates its nuclear translocation in mESCs. siRNA mediated ERα knockdown eliminates the promotive effects of Icaritin on the cell cycle regulators and key pluripotency transcription factors in mESCs, suggesting that the enhanced mESCs expansion capacity by Icaritin is at least partially mediated by its regulation on ERα.

## Results

### Icaritin enhances self-renewal of mESCs in association with upregulation of core pluripotency transcription factors

**Figure 1:**
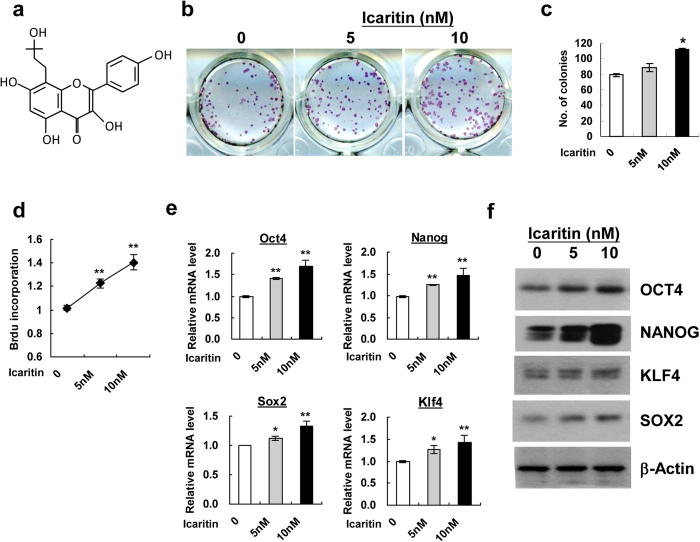
Icaritin promotes mESCs self-renewal. (**a**) The chemical structure of Icaritin. (**b**) mESCs were exposed to 5 nM or 10 nM Icaritin for 5 days, undifferentiated cell colonies were visualized by positive staining with ALP. (**c**) Quantitation of ALP^+^ colony numbers in (**b)**. (**d**) The mESCs proliferation was examined by BrdU incorporation assay following Icaritin exposure for 48 h. (**e**) mRNA expression of pluripotency transcription factors Oct4, Nanog, Sox2, Klf4 upon Icaritin treatment detected by quantitative real-time PCR. Expression levels were normalized to β-actin. (**f**) Protein levels of OCT4, NANOG, SOX2, KLF4 in mESCs after Icaritin treatment were detected by Western blot. β-actin was used as a loading control. Shown are cropped from the original blots. Values were the mean ± SD (n = 3). *P < 0.05; **P < 0.01.

The self-renewal of mESCs is characterized by the ability of infinite proliferation while maintaining an undifferentiated state. To determine the role of Icaritin in regulating mESCs self-renewal, we seeded mESCs at low density and performed colony formation assay with addition of Icaritin (Fig. 1a) at different concentrations for 5 days. Undifferentiated cells were visualized by positive staining of alkaline phosphatase (ALP^+^) in mESC colonies. Icaritin treatment induced a significant increase in ALP^+^ colony numbers, where there were 25% more ALP^+^ colonies in mESCs cultures upon 10 nM Icaritin exposure than that of the controls (Fig. 1b,c). mESCs also exhibited a 20% and 40% increase in the BrdU incorporation after 5 nM and 10 nM Icaritin exposure for 48 h respectively (Fig. 1d), indicating enhanced proliferation rate of mESCs by Icaritin. In the meantime, real-time PCR and Western blot analysis revealed an upregulation of mRNA and protein expression of core pluripotency transcription factors OCT4, NANOG, KLF4 and SOX2 in mESCs by Icaritin compared with none treatment control respectively (Fig. 1e,f). These results suggested that Icaritin exerted a positive effect on mESCs proliferation generating undifferentiated colonies in association with elevation of key transcription factors controlling pluripotency.

### Prolonged Icaritin exposure sustains mESCs pluripotency

**Figure 2:**
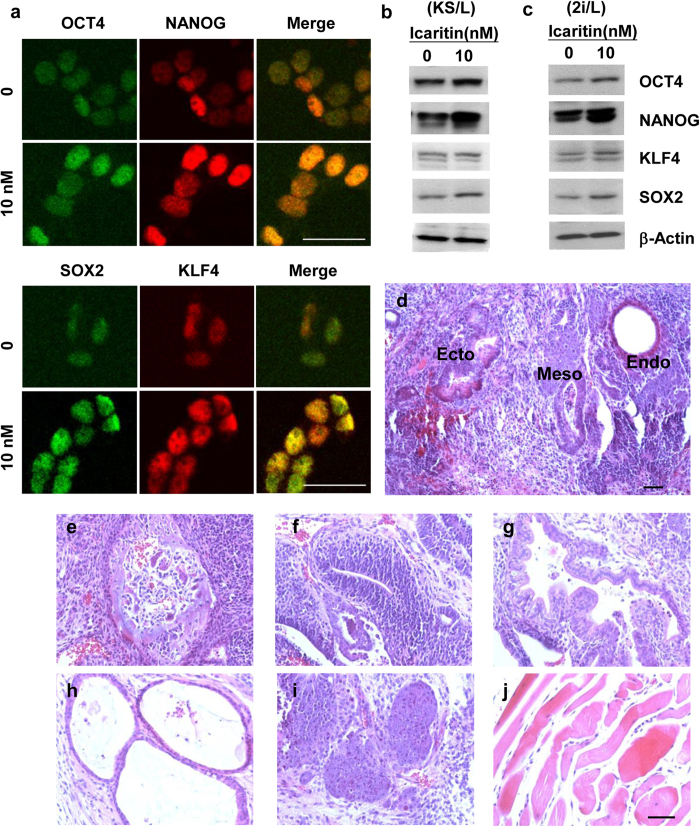
Long-term culture with Icaritin sustains mESCs pluripotency. mESCs were maintained in 10 nM Icaritin for 5 passages. Pluripotency transcription factors expression was detected by immunofluorescence staining (**a**) and Western blot analysis (**b**) respectively. Pluripotency transcription factors expression for mESCs cultured in 2i/LIF (2i/L) condition with or without Icaritin exposure was also examined (**c**). Representative images and cropped bands from the original were shown. 0 nM, none treatment control; 10 nM, mESCs maintained in culture media supplemented with 10 nM Icaritin for 5 passages. (**d**–**j**) Hematoxylin & Eosin staining of teratoma sections obtained from subcutaneous transplantation of long-term Icaritin-treated mESCs in SCID mice (n = 4). (**d**) Examination of differentiated tissues derived from three germ layers in a lower magnification. Representative images in higher magnification of differentiated tissues including (**e**) Bone, (**f**) Rosette of neural epithelium, (**g**) gut-like epithelium, (**h**) respiratory like epithelium, (**i**) Epidermis and (**j**) Muscle were shown. Scale bars = 100 μm.

We next examined whether the pluripotency of mESCs could be sustained in long-term culture supplemented with Icaritin. mESCs were maintained in complete medium containing 10 nM Icaritin for 5 passages. Compared with mESCs without drug exposure, Icaritin significantly increased the protein expression of OCT4, NANOG, SOX2 and KLF4 as indicated by the confocal microscopy showing increased fluorescent intensity in the cell nuclei stained with respective specific antibodies (Fig. 2a). This observation was in consistency with an upregulation of the corresponding proteins as detected by Western blot (Fig. 2b). We next examined whether Icaritin has the similar effect on mESCs when cultured in the defined serum free medium containing CHIR99021 and PD0325901 (2i/LIF medium), as 2i/LIF was defined as a powerful condition for maintaining mESCs self-renewal^20^. Interestingly, Icaritin was capable to upregulate pluripotency transcription factors in mESCs like those cultured in knockout serum/LIF conditions (Fig. 2c). The data suggest that Icaritin activates core pluripotency factors in mESCs in both prime and naïve states. To further verify the pluripotency of Icaritin-treated mESCs *in vivo*, the expanded mESCs were transplanted subcutaneously into the dorsal flank of severe combined immunodeficiency (SCID) mice for teratoma formation. 6 weeks later, Icaritin-treated mESCs formed the teratoma and differentiated into tissues of all the three germ layers: ectoderm, endoderm and mesoderm (Fig. 2d). Various differentiated tissue types including bone (Fig. 2e), neural epithelium (Fig. 2f), gut-like epithelium (Fig. 2g), respiratory-like epithelium (Fig. 2h) epidermis (Fig. 2i) and muscle (Fig. 2j) were observed in the sections of the teratoma. Taken together, an increased ALP^+^ colony formation of Icaritin treated mESCs and their long term maintained pluripotency indicated the potency of Icaritin on promoting mESCs self-renewal.

### Icaritin promotes G1/S phase progression through modulation of cell cycle machinery

**Figure 3:**
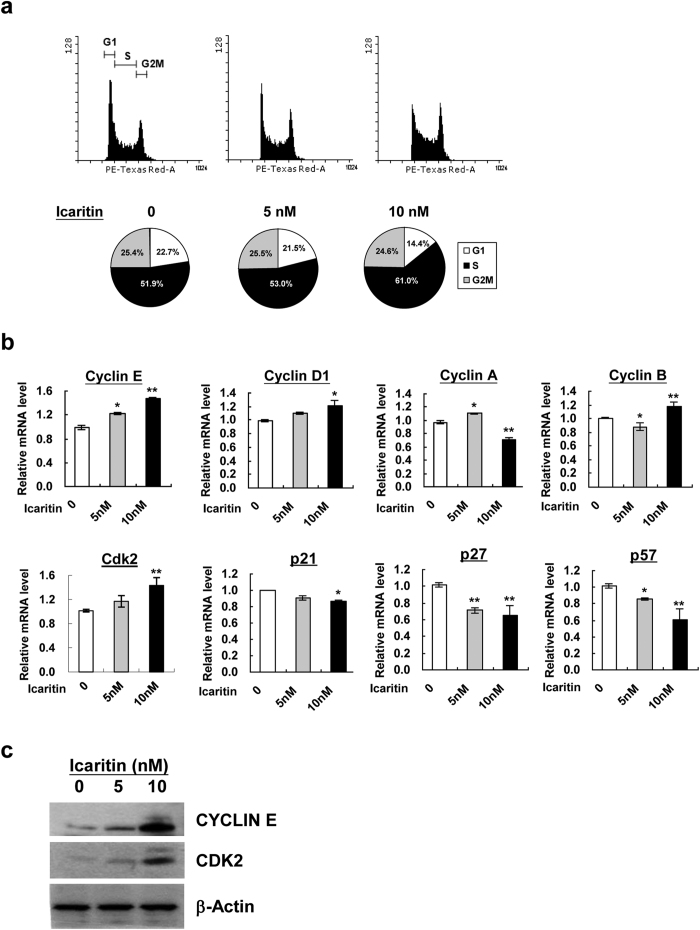
Icaritin participates in regulation of cell cycle dynamics and regulatory machinery. (**a**) The percentages of mESCs populations in G1, S and G2/M phases were analyzed by flow cytometry. 10000 cells were counted in each sample. (**b**) mRNA expression of Cyclin E, Cyclin D1, Cyclin A, Cyclin B, Cdk2, p21, p27 and p57) was detected by quantitative real-time PCR. Values were the mean ± SD (n = 3). *P < 0.05; **P < 0.01. (**c**) Detection of Cyclin E and CDK2 protein levels in mESCs after Icaritin treatment by Western blot analysis. β-actin was used as a loading control. Shown are cropped from the original blots.

Rapid cell division of self-renewing mESCs exhibits a unique cell cycle dynamics as characterized by a short G1 phase and a high proportion of cells in S phase, which favors an effective DNA replication and nuclear division^22^. As the molecular mechanisms of cell cycle control for ESCs have been functionally linked with the stemness of ESCs, we speculate that Icaritin-enhanced self-renewal potential may relate to a change in the cell cycle dynamics. To support this notion, the cell cycle profile of mESCs following Icaritin treatment was examined by flow cytometric quantitation of DNA content stained with propidium iodide. Intriguingly, Icaritin exposure increased the percentage of cell population in S phase accompanied by a reduction of the cell proportion in G1 phase (Fig. 3a). The result indicated a G1 to S phase shift of cell cycle dynamics in mESCs following Icaritin treatment. To elucidate whether the change in cell cycle pattern was associated with alterations of cyclins and cyclin-dependent kinase inhibitors (CKIs), the respective mRNA transcript levels in mESCs with and without Icaritin treatment were examined by quantitative real-time PCR. A dose-dependent increase in Cyclin E and Cyclin D1 transcripts as well as a down-regulation of CKIs including p21, p27 and p57 in mESCs was observed following Icaritin treatment (Fig. 3b). Cyclin A and Cyclin B mRNA expression appeared in variance with different dosages of Icaritin (Fig. 3b). Notably, there was about 20% and 40% increase in Cyclin E transcripts upon 5 nM and 10 nM Icaritin exposure respectively, which was accompanied by upregulation of Cdk2 transcript level (Fig. 3b). The phenotype was in accord with a remarkable upregulation of the protein expression of Cyclin E and its specific catalytic subunit CDK2 in a dose-dependent manner (Fig. 3c). As Cyclin E/CDK2 complex is a major regulatory element for cell cycle progression from G1 to S phase, the promotional entry of S phase of cell cycle by Icaritin might correlate with the increased expression of Cyclin E and CDK2 in mESCs.

### Icaritin modulates the expression profile of genes involved in stem cell maintenance of mESCs

**Figure 4:**
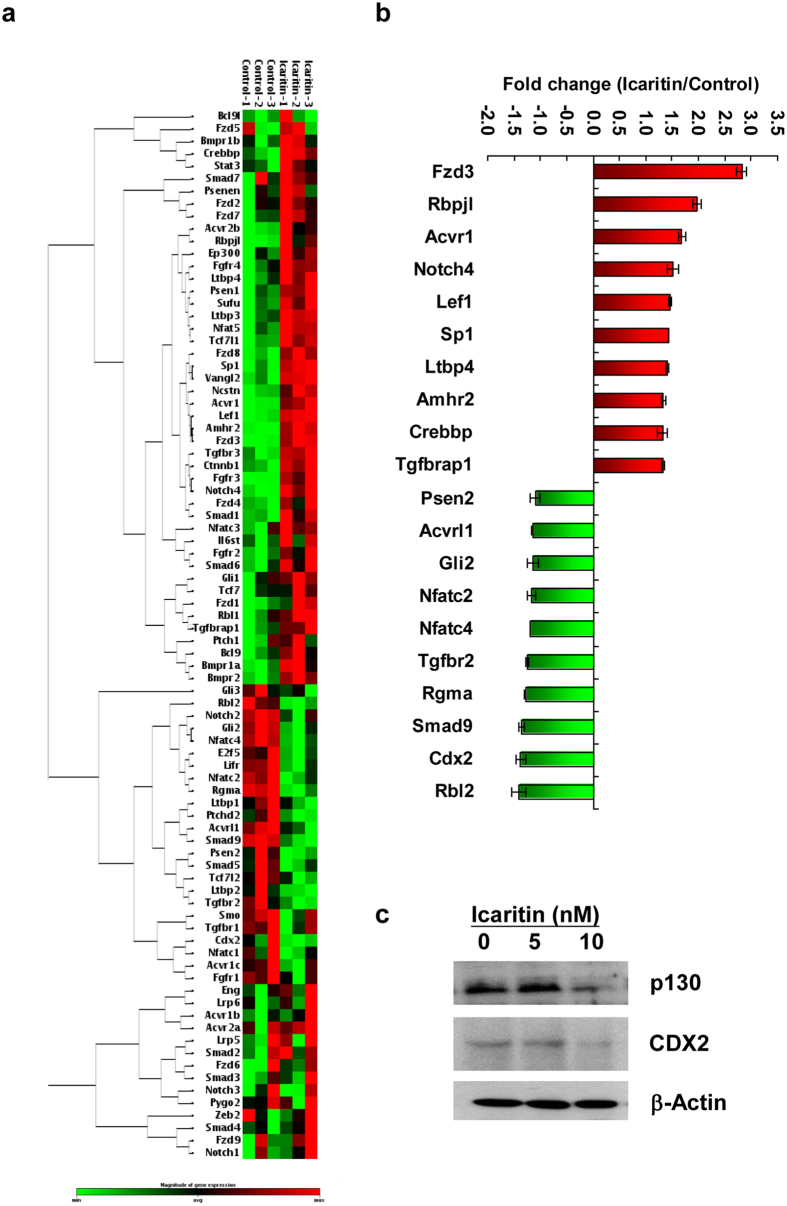
Icaritin modulates the expression profile of genes in relation to stem cell signaling. mESCs were exposed to 10 nM Icaritin for 24 h. Total RNA was extracted, reverse transcribed and amplified in PCR arrays as indicated in Materials and Methods. The experiment was performed in triplicates. (**a**) Hierarchical clustering analysis on the PCR array data. (**b**) A summary of top ten up-regulated and down-regulated genes in mESCs following Icaritin treatment compared with the controls. (**c**) Protein levels of p130 and CDX2 after Icaritin treatment were examined by Western blot analysis. β-actin was used as a loading control. Shown are cropped from the original blots.

To further examine the underlying mechanism for Icaritin-induced self-renewal of mESCs, the expression profile of 84 genes involved in pluripotency maintenance and other signal transduction pathways of fibroblast growth factor (FGF), Hedgehog, Notch, TGF-beta and Wnt was compared by performing PCR array analysis (Supplementary Table 2). 14 genes were found to be up-regulated in response to Icaritin exposure, including frizzled homolog 3 (Fzd3, 2.83-fold), recombination signal binding protein for immunoglobin kappa J-like (Rbpjl, 1.96-fold) and activin A receptor Ic (Acvr1c, 1.69-fold) (Supplementary Table 2). In contrast, 10 genes were shown to be significantly down-regulated. Retinoblastoma-like 2 (Rbl2 or p130) was remarkably suppressed by 1.41-fold in response to Icaritin treatment, and Cdx2 and Smad9 were decreased by 1.37-fold and 1.36-fold respectively (Supplementary Table 2). Hierarchical clustering of genes from PCR array and the top-ten up or down-regulated genes upon Icaritin exposure was shown in Fig. 4a and b respectively. Although the 24 differentially expressed genes were mainly enriched in the FGF signaling pathway and TGF-beta superfamily signaling pathway, they also distributed in the Hedgehog, Wnt and Notch signaling pathways, indicating that Icaritin possesses biological or pharmacological effects on mESCs through multiple pathways and those pathways may function in a coordinated manner in promoting mESCs self-renewal. As noted that Icaritin exhibited a significant impact on G1/S transition of mESCs (Fig. 3a), we then specifically investigated CDX2 and p130 (Rbl2 gene product) that involved in the TGF-beta and FGF signaling pathways respectively. Both proteins have been indicated to participate in cell cycle regulation in ESCs or cancer cells^23^,^24^,^25^. The decreased expression of CDX2 and p130 in mESCs especially following 10 nM Icaritin exposure was validated by Western blot analysis (Fig. 4c), which coincided with the corresponding transcript suppression in mESCs in response to Icaritin treatment compared with the none treatment controls (Fig. 4b).

### Inhibition of CDX2 and p130 enhances mESCs self-renewal mediated by coordinated upregulation of Cyclin E/CDK2 and pluripotency transcription factors

**Figure 5:**
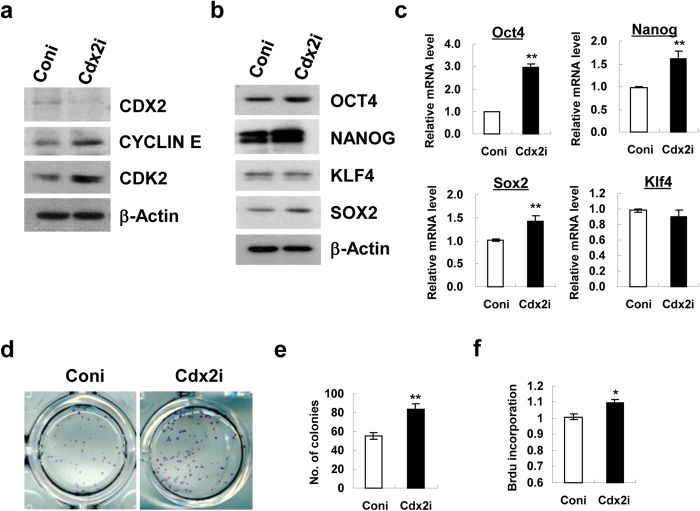
CDX2 regulates the expression of Cyclin E/CDK2 and pluripotency transcription factors in mESCs. mESCs were transfected with control (Coni) or Cdx2 (Cdx2i) siRNA. (**a**) Protein levels of Cyclin E and CDK2 in response to Cdx2 gene knockdown were detected by Western blot analysis. Protein (**b**) and mRNA (**c**) expression of OCT4, NANOG, SOX2, KLF4 following Cdx2 knockdown was examined by Western blot and quantitative real-time PCR respectively. Western blot shown are cropped from the original blots. (**d**) Representative images of ALP^+^ colonies at day 5 following siRNA mediated Cdx2 knockdown in mESCs. (**e**) Quantitation of ALP^+^ colonies at day 5 following Cdx2 knockdown. (**f)** BrdU incorporation assay for cell proliferation following siRNA mediated Cdx2 knockdown in mESCs. Each treatment was performed in triplicate. Values were the mean ± SD (n = 3). *P < 0.05; **P < 0.01.

We next determined whether Cdx2 or p130 gene knockdown through siRNA transfection could reproduce the observed phenotypes in mESCs after Icaritin treatment. Knockdown of Cdx2 gene resulted in an elevation of protein levels for Cyclin E and CDK2 in mESCs (Fig. 5a). This was accompanied by an upregulation in both mRNA and protein levels of OCT4, NANOG and SOX2 (Fig. 5b,c). To test the biological effect of Cdx2 knockdown in the self-renewal capacity of mESCs, the cells were re-plated after siRNA transfection and undifferentiated mESC colonies were visualized by ALP staining. A significant increase (50%) in the number of ALP^+^ colonies was observed following Cdx2 gene suppression as compared with control siRNA group (Fig. 5d,e). In addition, Cdx2-knockdown mESCs showed increased proliferation rate indexed by BrdU incorporation assay than that of the control siRNA group (Fig. 5f).

**Figure 6:**
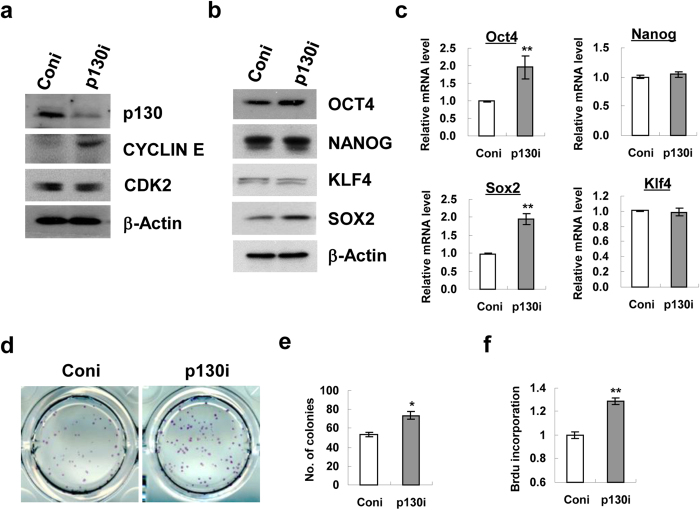
p130 modulates the expression of Cyclin E and pluripotency transcription factors. mESCs were transfected with control (Coni) or p130 (p130i) siRNA followed by further analysis. (**a**) Protein levels of Cyclin E and CDK2 in response to p130 gene knockdown was detected by Western blot analysis. Protein (**b**) and mRNA (**c**) expression of OCT4, NANOG, SOX2, KLF4 in mESCs following p130 knockdown was examined by Western blot and quantitative real-time PCR respectively. Western blot shown are cropped from the original blots. (**d**) Representative images of ALP^+^ colonies at day 5 following siRNA mediated p130 knockdown in mESCs. (**e)** Quantitation of ALP^+^ colonies at day 5 following p130 knockdown. (**f**) BrdU incorporation assay for cell proliferation following siRNA mediated p130 knockdown in mESCs. Each treatment was performed in triplicate. Values were the mean ± SD (n = 3). *P < 0.05; **P < 0.01.

Similar but distinct phenotypes were observed in mESCs following siRNA mediated knockdown of p130. Decreased expression of p130 caused an up-regulation of Cyclin E but not CDK2 protein level in mESCs (Fig. 6a). Furthermore, an increase in mRNA and protein expression of OCT4 and SOX2 was observed while there was no apparent change in Nanog and KLF4 expression in mESCs after p130 siRNA transfection (Fig. 6b,c). The ALP^+^ colony numbers in mESCs cultures were increased by 25% after p130 knockdown than that of the control siRNA group (Fig. 6d,e), which was accompanied by an increase in proliferation rate in the BrdU incorporation assay (Fig. 6f).

### Icaritin interacts with ERα and regulates its nuclear translocation in mESCs

**Figure 7:**
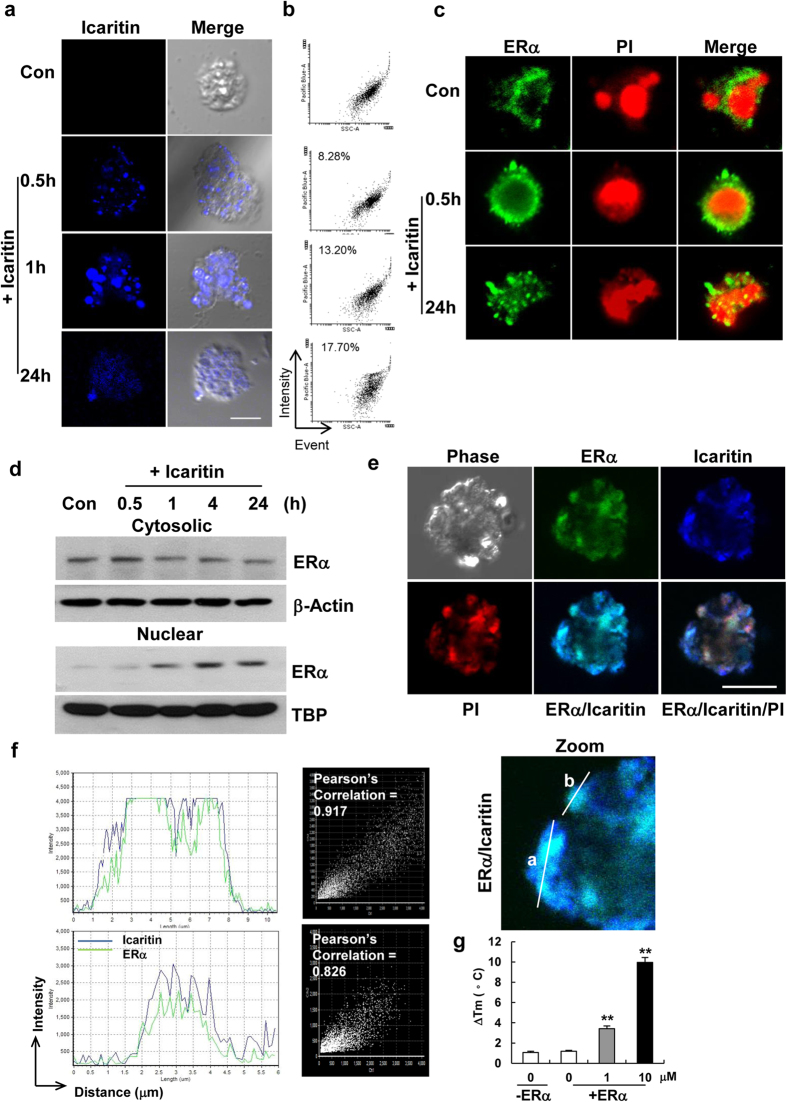
ERα nuclear translocation and colocalization with Icaritin. Icaritin uptake by mESCs at the indicated time points was detected by confocal microscopy using excitation wavelength 350 nm (**a**) and flow cytometric analysis (**b**) respectively. (**c**) Translocation of ERα protein into the nucleus was visualized by immunostaining with ERα antibody and nuclear counterstain by PI. (**d)** Time-dependent ERα protein nuclear translocation was detected by Western blot analysis of protein extracted from cytosolic and nuclear fractions. Shown are cropped from the original blots. (**e)** Representative immunofluorescent images showing colocalization of Icaritin and ERα protein in mESCs upon 24 h of drug exposure. (**f)** Representative Icaritin/ERα colocalization (line a and b) in mESCs shown in (**e**). Line profiles indicated the extent of colocalization over distance. Average Pearson’s Correlation analyses indicated high levels of colocalization of ERα and Icaritin. (**g**) A direct interaction of Icaritin with ERα was validated by protein thermal shift assay. Experiment was performed in the absence or presence of ERα recombinant protein with an indicated dose of Icaritin. The resulting ΔTm values were analyzed by thermal protein shift software. Values were the mean ± SD (n = 4). **P < 0.01.

Under confocal microscopy, Icaritin was found to emit blue fluorescent signal when excitation wave length at 350 nm. Apparent Icaritin uptake by mESCs, shown by an accumulation of intracellular fluorescent signal, was observed at 30 min after drug exposure, and the signal was distributed throughout the whole cells following 24 h incubation (Fig. 7a). Icaritin uptake was then further quantified by flow cytometric analysis. An increase in percentage of fluorescent intensity from the same number of mESCs indicated that Icaritin uptake by the cells was increased with prolonged incubation time (Fig. 7b). We next examined whether Icaritin uptake was associated with ligand-induced changes in signal transduction. Interestingly, ERα immunofluorescence staining revealed a time-dependent ERα nuclear translocation in mESCs induced by Icaritin (Fig. 7c). A simultaneous decrease of cytosolic ERα and an upregulation of nuclear ERα expression were also detected by Western blot analysis (Fig. 7d). Moreover, merging of fluorescence image of Icaritin and ERα expression indicated multiple cellular localization sites throughout the mESCs colony (Fig. 7e). A high degree of colocalization of signals of Icaritin and ERα was further proven by Average Pearson’s Correlation analysis (Fig. 7f). The *bona fide* interaction of Icaritin with ERα was validated by performing fluorescence-based thermal shift assay. The assay works on the fact that ligand binding alters thermal stability of proteins, indicated by a shift in the resulting melting temperature. Melting reactions from Icaritin alone or ERα recombinant protein alone induced a limited temperature shift (less than 2 °C), while a significant increase in thermal shift with ΔTm of 3.7 °C was observed from a reaction of 1 μM Icaritin with ERα. A greater magnitude of thermal shift with ΔTm of 10.08 °C occurred at 10 μM Icaritin (Fig. 7g). The results indicated that a direct physical interaction of Icaritin and ERα existed in mESCs when treated with Icaritin.

### Icaritin-ERα direct interaction is essential for cellular uptake of Icaritin and upregulation of mESC pluripotency transcription factors

**Figure 8:**
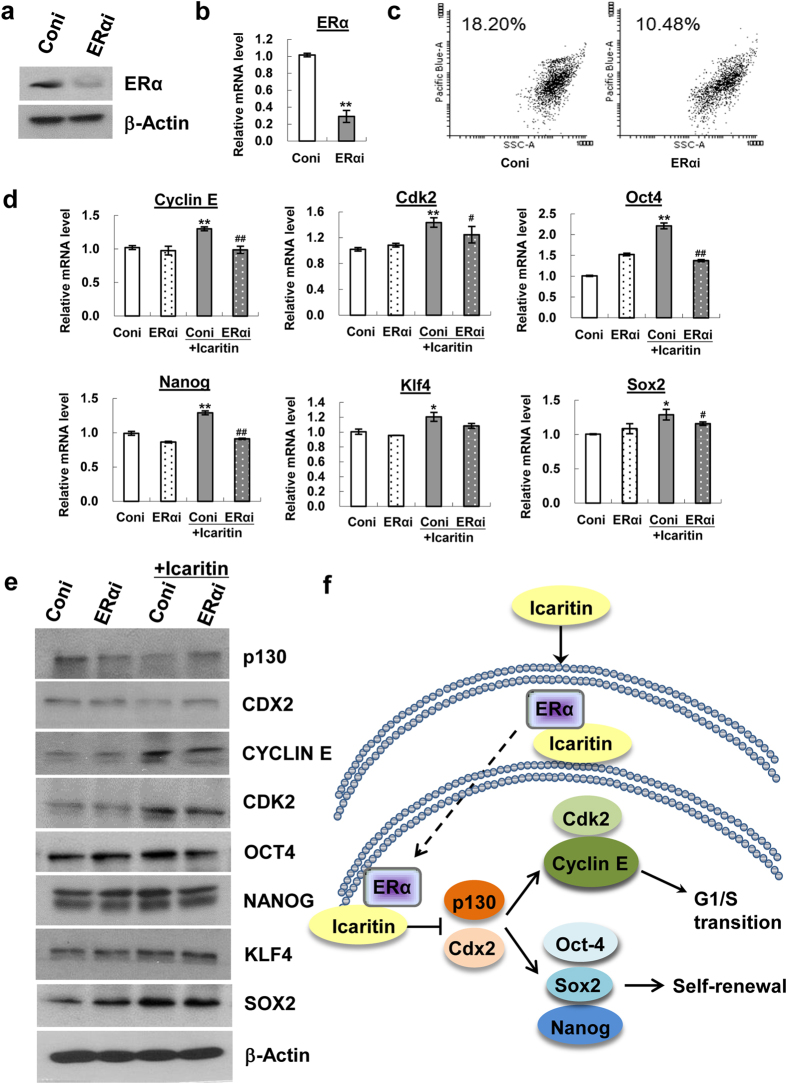
ERα is required for Icaritin uptake and mediates the promotive effects of Icaritin on the expression of pluripotency transcription factors of mESC. mESCs were transfected with control siRNA (Coni) or ERα siRNA (ERαi) followed by further analysis. The ERα protein (**a**) and transcript level (**b**) after transfection was verified by Western blot and real-time PCR respectively. Values were the mean ± SD (n = 3). **P < 0.01. (**c**) The effect of ERα knockdown on Icaritin cellular uptake was examined by flow cytometric analysis. Cells were collected at 24 h after Icaritin exposure. mRNA (**d**) and protein (**e**) levels of selected cell cycle regulators and pluripotency transcription factors in response to ERα knockdown with or without Icaritin treatment was detected by real-time PCR and Western blot analysis respectively. Western blot shown are cropped from the original blots. Values were the mean ± SD (n = 3). ^#^*P* < 0.05, ^##^*P* < 0.01, compared with control siRNA under Icaritin treatment. *P < 0.05, **P < 0.01, compared with control siRNA without Icaritin treatment. (**f**) Proposed model of functional role of Icaritin in promoting mESCs self-renewal. Icaritin interacts and activates ERα, functions as a potent inhibitor for CDX2 and p130 to activate Cyclin E/CDK2 signaling and upregulate core-pluripotency transcription factors expression, subsequently promote G1/S phase transition and self-renewal of mESCs.

To determine whether a physical binding of Icaritin with ERα is functional for enhanced self-renewal phenotype, mESCs were transfected with ERα siRNA to knockdown ERα expression, and an efficient knockdown of ERα at mRNA and protein levels was achieved compared with that of the control siRNA (Fig. 8a,b). ERα knockdown remarkably suppressed Icaritin uptake compared with that of the control siRNA treated cells (18.2% vs 10.48%) detected by flow cytometric analysis at 24 h post-transfection (Fig. 8c). The results suggested that ERα expression and the Icaritin-ERα interaction played a critical role in Icaritin uptake by mESCs. Furthermore, ERα knockdown attenuated the suppressive effect of Icaritin on p130 and CDX2 expression as well as blocked its promotive effect on Cyclin E, CDK2 and pluripotency transcription factors expression including Oct4, Nanog, Klf4 and Sox2 (Fig. 8d,e). This data was in consistent with a decrease in intracellular Icaritin level. Our results suggest that Icaritin directly interacts with ERα, and induces ERα nuclear translocation, where it suppresses the expression of CDX2 and p130, upregulates Cyclin E/CDK2 signaling and core pluripotency transcription factors OCT4, NANOG, KLF4 and SOX2, subsequently modulates the S phase progression and self-renewal of mESCs (Fig. 8f).

## Discussion

Elucidation of the molecular control of ESCs self-renewal and optimization of the culture conditions for ESCs maintenance and expansion are the keys to potential application of ESCs for drug screen and regenerative therapy. In this study, we identify Icaritin, a phytoestrogen molecule, interacts and activates ERα, functions as a potent inhibitor for CDX2 and p130, which subsequently activates Cyclin E/CDK2 signaling to promote G1/S phase progression and up-regulates the expression of OCT4, NANOG, KLF4 and SOX2, resulting in a contribution to long-term expansion of mESCs while maintaining their pluripotency.

Icaritin is an active component of flavonoid extracted from *Herba Epimedii*, a commonly prescribed Chinese herb medicine. It is indicated that Icaritin possesses a large panel of biological and pharmacological activities, including induction of cardiac differentiation of mESCs^26^,^27^, neuronal differentiation and protection^28^,^29^, promoting osteogenic differentiation while suppressing adipogenesis and osteoclastogenesis^30^,^31^, as well as anti-cancer activities^32^,^33^,^34^. Icaritin increases the proliferation of estrogen receptor (ER)-positive or negative human breast cancer cells through distinct signaling pathways^33^,^35^. Taken the fact that both cancer cells and ESCs proliferate indefinitely, they might share some common molecular mechanisms of cell cycle control. Intriguingly, in our screening assay we observe that Icaritin increases mESCs proliferation and ALP^+^ colony formation (Fig. 1b–d), as well as sustains the pluripotency phenotype of mESCs in the teratoma formation model after long-term treatment (Fig. 2d–j). This suggests that supplementation of Icaritin in traditional mESCs culture media could be used as an approach to enhance mESCs self-renewal and expansion.

mESCs are featured by the establishment of an unique cell cycle program which allows rapid cell proliferation, with a relatively short G1 phase and a high proportion of cells in S-phase^21^. On the contrary, differentiating mESCs display a dramatic change in cell cycle dynamics, particularly an expansion of G1 phase^36^. Recent studies on hESCs also reveal that cell fate decision is tightly controlled by the cell-cycle positions^37^ and cells in the G1 phase exhibit elevated expression of developmental regulators and are more susceptible to differentiation signals^38^. Therefore, S phase dynamics in the cell cycle of pluripotent stem cells is essential to maintain the self-renewal phenotype. In this study, a remarkable G1 to S phase progression in mESCs following Icaritin treatment (Fig. 3a) and the activation of Cyclin E/CDK2 as well as down-regulation of CKIs including p21, p27 and p57 (Fig. 3b,c) imply that Icaritin may modulate the cell cycle position to control self-renewal of mESCs. Cyclin E is a well-known cyclin in control of G1/S phase transition in complex with CDK2^39^. And CKIs are regarded as negative regulators of cell cycle progression and inhibit self-renewal regulators to induce differentiation^40^. Of note, Cyclin E-CDK2 activity is periodic in somatic cells but is constitutively active in ESCs. This makes ESCs effectively bypass the restriction point which separates early G1 from late G1^41^. On the other hand, cell cycle can be lengthened or arrested in G1 phase by overexpression of CKIs. An induction of Cyclin E-CDK2 basal expression and/or downregulation of CKIs by Icaritin may favor to enhance the process of G1-S phase transition, resulting in increased cell proliferation. However, it is interesting to note that Icaritin (10 μM) induces apoptotic cell death in human endometrial cancer Hec1A cells, with marked decrease of Cyclin D1 and increase of p21 and p27 expression^32^. In another study, Icaritin (up to 10 μM) does not induce cell death but instead stimulates dose-dependent osteogenic differentiation in mesenchymal stem cells^42^. In the present study, a much lower dosage of Icaritin (10 nM) was employed in mESCs culture. The discrepancy of Icaritin effects on different cell types indicates that the role of Icaritin on cellular function might be dosage or cell type dependent. The regulation of cell cycle dynamics during the Icaritin-induced mESCs self-renewal is further supported by the findings that two cell cycle regulators CDX2 and p130 are dramatically down-regulated in mESCs following 10 nM Icaritin treatment (Fig. 4). Suppression of CDX2 is found to stimulate G1/S phase transition associated with low levels of p27 and activation of cyclin E/CDK2 in mice, in human colon cancer cell line and rat intestinal epithelial cell line^43^. p130 and other RB family members including p107 and pRB are well-known for their ability to restrict G1/S transition through binding and repressing E2F transcription factor^44^. Our study shows that siRNA mediated knockdown of Cdx2 results in the elevated protein levels of Cyclin E and CDK2 (Fig. 5a), which is comparable to that observed in mESCs treated with Icaritin (Fig. 3c). In contrast to pRB, p130 is able to bind and directly inhibit Cyclin E/CDK2 in regulation of S phase entry and M phase exit^45^. p130 knockdown up-regulates Cyclin E expression only (Fig. 6a). Besides the effects on Cyclin E/CDK2 complex implicated in G1/S phase progression, knockdown of Cdx2 or p130 reproduces most of the phenotypes that have been observed in mESCs treated with Icaritin including the up-regulation of OCT4, SOX2 or NANOG, enhanced cell proliferation, and increased ALP^+^ colony numbers (Fig. 5b–f and Fig. 6b–f). Therefore, CDX2 and p130 serve as important regulators that are inhibited by Icaritin to promote G1/S transition and enhance mESCs self-renewal, though other regulators and pathways may also be targeted by Icaritin and function independently or coordinately with CDX2 and p130. On the other hand, it is documented that during cell cycle transition, both CDX2^25^,^46^ and RB protein^23^,^47^ can be phosphorylated by CDK2-associated complexes resulting in rapid degradation or inactivation. Thus, it would be possible that a feedback loop exists in Icaritin treated mESCs in which the increased Cyclin E level or elevated CDK2 kinase activity caused by the inhibition of CDX2 and p130 may further decrease the expression levels of CDX2 and p130, to favor G1/S phase progression and mESCs self-renewal.

G1/S phase progression in mESCs following Icaritin treatment is accompanied by significant increase in the core pluripotency transcription factors. This could be a direct consequence of the inhibition of CDX2 or p130 by Icaritin. Indeed, previous study shows that CDX2 can directly bind to the Oct4 promoter to inhibit its transcription, leading to ESCs differentiation into trophectoderm^48^. However, it deserves further investigating whether CDX2 or p130 targets at multiple core pluripotency transcription factors at the same time, or they may only directly modulate one single target and the combined up-regulation in OCT4, SOX2 and NANOG could be a result from the interdependent transcriptional regulatory networks among those factors^49^,^50^. In addition, our PCR array analysis reveals that Icaritin treatment alters the expression of genes from multiple stem cell signaling pathways. These findings may constitute part of a complex stem cell self-renewal regulation model that awaits further investigation.

In contrast to a remarkably downregulation of CDX2 and p130 in mESCs following Icaritin treatment, Fzd3 and Rbpjl are the most two up-regulated genes in the PCR array. Fzd3, a member of frizzled receptor family, acts as one of the receptors for Wnt proteins. Most of the frizzed receptors are coupled to canonical Wnt/β-catenin signaling pathways. Wnt signaling plays an important role in the balance between progenitor self-renewal and differentiation. It is reported that overexpression of certain Wnt ligands supports self-renewal of progenitor cells^51^,^52^. However, little is known about how Wnt receptors involve in stem cell maintenance. In human ESCs, Fzd7 is elevated in undifferentiated cells relative to differentiated cell populations. In contrast, the pluripotent state of human ESCs is disrupted by blocking FZD7 expression^53^. Upregulation of Fzd3 in mESCs in response to Icaritin treatment indicates that Fzd3 may play some role in facilitating the activation of Wnt signaling in promoting mESCs self-renewal. Rbpjl is regarded as an exocrine transcription factor and a component of heterotrimeric transcriptional complex PTF1, which participates in expression of secretory digestive enzymes during pancreatic acinar cell development^54^. Transduction of Rbpjl gene in mESCs enhances acinar differentiation for maximal production of digestive enzymes^55^. Though Rbpjl in mESCs is upregulated by Icaritin, it is not known whether Rbpjl plays a role in mESCs self-renewal. The detailed molecular mechanisms of Fzd3 and Rbpjl in regulating mESCs self-renewal deserve to be further defined.

Another striking phenotype we observe is that Icaritin treatment remarkably induces ERα nuclear translocation in mESCs examined by confocal microscopy and Western blot analysis (Fig. 7c,d). This suggests that, as a phytoestrogen molecule, Icaritin may function as a ligand or agonist to ERα. Thus, we speculate that Icaritin might structurally or physically interact with ERα and regulate its function. Indeed, a high degree of Icaritin and ERα colocalization is observed at subcellular locations of mESCs (Fig. 7e,f). This phenomenon is further characterized by a fact that Icaritin elicites a dosage dependent thermal shift of ERα protein (Fig. 7g), indicating the physical interaction of Icaritin and ERα. In fact, protein thermal shift assay has been widely used as a reliable tool in high throughput screening of drug target identification^56^, ^57^, ^58^. Next we prove that ERα knockdown significantly decreases cellular uptake of Icaritin (Fig. 8a–c), and attenuates the promotive effects of Icaritin on the expression of Cyclin E, Cdk2, Oct4, Nanog, Klf4 and Sox2 (Fig. 8d,e). Therefore, Icaritin-ERα direct interaction is functionally essential for Icaritin uptake and regulation of cell cycle signals and core pluripotency transcription factors of mESCs.

Taken together, Icaritin functions as a potent inhibitor for Cdx2 and p130 in mESCs, which activates Cyclin E/CDK2 signaling and elevates core pluripotency transcription factors expression, subsequently integrates the cell cycle control and transcriptional regulatory networks to enhance mESCs self-renewal. These effects are at least partially mediated by its physical interaction and activation of ERα. The results provide evidence for potential application of Icaritin as culture medium supplement for expansion of pluripotent stem cells.

## Methods

### Cell culture

The D3 mESCs were cultured on a feeder layer of mitomycin-treated MEFs in a traditional complete medium (KS/LIF medium) containing knockout Dulbecco’s modified Eagle’s medium (DMEM/F-12) supplemented with 15% knockout serum replacement, 0.1 mM non-essential amino acids, 1% penicillin/streptomycin, 1 mM L-glutamine, 0.1 mM β-mercaptoethanol (Invitrogen) and 1000 U/ml mouse LIF (Millipore). 2i/LIF condition medium was made from the traditional complete medium by using 3 μM CHIR99021 (Stemgent) and 1 μM PD0325901 (Stemgent) to substitute knockout serum replacement. Prior to experiments, cells were passaged by incubation in 0.25% trypsin/EDTA for 5 min at 37 °C and then plated onto gelatin-coated dishes without a feeder layer for 24 h. Icaritin was purchased from Sigma. All reagents were purchased from Invitrogen unless otherwise specified.

### Transfection of siRNA oligos

mESCs were transiently transfected with siRNAs specific for knockdown of Rbl2/p130, Cdx2 or ERα gene (Santa Cruz). Cells were plated on gelatin coated plates for 24 h. Transfection was preceded by using lipofectamine RNAiMAX reagent according to manufacturer’s instructions (Invitrogen). Control siRNA transfection was performed in parallel. Total RNA and protein was extracted at 48 h after transfection.

### BrdU incorporation assay

The proliferation of mESCs was examined by the measurement of BrdU incorporation into the newly synthesized DNA using Cell Proliferation ELISA Kit, BrdU (Colorimetric) (Roche). Briefly, the cells were washed, fixed and labeled with BrdU for 4 h. Thereafter the cells were incubated with peroxidase-conjugated anti-BrdU antibody for 90 min. BrdU incorporation was detected by incubating the cells with tetramethyl-benzidine (TMB) as a substrate. The color development, which was directly proportional to the amount of DNA synthesis and hereby to the number of proliferating cells, was quantified by measuring the absorbance at 370 nm by a microplate reader.

### Colony formation assay and alkaline phosphatase (ALP) staining

250 mESCs were seeded on gelatin-coated 24-well plates for 24 h and exposed to complete medium containing 5 nM or 10 nM Icaritin (Sigma) for 5 days. Undifferentiated colonies characterized by the expression of ALP were detected by staining with ALP detection kit (Millipore) following manufacturer’s instructions. Each sample was performed in triplicates.

### Quantitative real-time PCR

Total RNA was extracted using the RNeasy Mini Kit (Qiagen). First strand cDNA was synthesized from 1 μg of total RNA in the presence of oligo-dT_12-18_ primer (Invitrogen) and 200 units of MMLV reverse transcriptase according to manufacturer’s protocol (Promega). Quantitative real-time PCR was performed using the SYBR Premix Ex Taq (Takara) in ABI Fast Real-time PCR 7900HT System (Applied Biosystems). Each reaction was performed in triplicates under the following parameters: 95 °C for 30 s and 40 cycles of 95 °C for 10 s. Target mRNA levels were normalized to β-actin mRNA expression. Primer sequences were listed in Supplementary Table 1.

### Western blot analysis

Following treatment with or without Icaritin, mESCs were washed three times with ice-old PBS. Total protein was extracted by cell lysis in Lammeli’s lysis buffer containing 1% Triton X-100, and scraped by a cell lifter. Protein concentration from the cell lysate was examined by BCA protein assay (Pierce). 30 μg protein was denatured, resolved in 12% SDS-PAGE minigel, and electrophoretically transferred onto an Immobilon-P membrane (Millipore). The membrane was pre-blocked with 5% nonfat dry milk for 1 h and hybridized with antibodies against OCT4, NANOG, SOX2, KLF4, p130, CDX2, Cyclin E and CDK2 (all from Cell Signaling), at 4 °C overnight with agitation. The membrane was washed extensively with 0.1% Tween-20 in PBS, and then incubated with horse-radish peroxidase conjugated secondary antibodies (Molecular Probes) in 1:8000 dilution at room temperature for 1 h. Protein expression signals were detected with enhanced chemiluminescence (GE Healthcare) and developed on an X-ray film (Roche).

### Teratoma formation assay

200,000 mESCs were resuspended in 30% Matrigel Basement Membrane Matrix (BD Bioscience) and injected subcutaneously into dorsal flank of severe combined immunodeficiency (SCID) mice. Four injections were performed in each mouse. After 6 weeks the mice were subjected for histology analysis for teratoma formation. The tumor like tissues were fixed with 10% formalin for 24 h and stored in 70% ethanol until paraffin embedding. 5 μm sections of the tissue were processed for Hematoxylin & Eosin staining. All experimental procedures were carried out by protocols approved by Animal Experimentation Ethics Committee from The Chinese University of Hong Kong and Animal (Control of Experiments) Ordinance from Department of Health, Hong Kong SAR. We confirmed that all methods were performed in accordance with the protocols approved by Animal Experimentation Ethics Committee from The Chinese University of Hong Kong and the regulations of (Control of Experiments) Ordinance from Department of Health, Hong Kong SAR.

### Immunofluorescence staining

mESCs seeded on gelatin-coated glass coverslip were washed with PBS, fixed with 4% paraformaldehyde (Sigma-Aldrich), permeabilized by 0.5% Triton X-100 for 10 min, and pre-blocked with 3% BSA for 1 h. Immunofluorescence staining was performed by incubation with OCT4, NANOG, SOX2 or KLF4 antibodies (all from Cell Signaling) at 4 °C overnight, followed by secondary antibodies Alexa-488 anti-mouse (OCT4 and SOX2) or Alexa-647 anti-rabbit (NANOG and KLF4) (Molecular Probes) for 1 h at room temperature. Cells were washed extensively with 0.1% tween-20 in PBS and mounted with DAPI (VectorLab). Fluorescent signal was visualized by Olympus FV1000 confocal microscope (Olympus). The image was processed by FV10-ASW software (Olympus).

### Flow cytometric analysis

Cells subjected to cell cycle analysis were trypsinized, fixed with 70% ethanol at 4 °C overnight and stained with propidium iodide in PBS at 37 °C for 30 min. For examination of Icaritin uptake, cells were trypsinized and washed twice with 1× PBS after being exposed to Icaritin at the indicated time points. Cell populations were analyzed by flow cytometry using LSRFortessa Analyzer (BD Bioscience). 10000 cells were counted in each sample. Data were analyzed with FACSDiva software.

### PCR array

Quantitative PCR array analysis was performed using mouse Stem Cell Signaling RT. Profiler PCR Array to examine the expression of 84 genes (Qiagen). mESCs were treated with or without 10 nM Icaritin for 24 h. Total RNA was extracted, reverse transcribed and PCR amplified according to manufacturer’s instructions. Data were analyzed using SABiosciences RT2 Profiler PCR Data Analysis software, available at http://pcrdataanalysis.sabiosciences.com/pcr/arrayanalysis.php.

### Protein thermal shift assay

The assay was performed according to manufacturer’s instructions (Invitrogen). Briefly, 1 μM or 10 μM Icaritin were mixed with 0.5 μg ERα recombinant protein (Abcam) in the presence of protein thermal shift dye up to a total volume of 20 μl. None drug control and none protein control were performed in parallel. All sample reactions were performed in four repeats. Protein melt reactions (25 °C for 2 min at 1.6 °C/s and 99 °C for 2 min at 0.05 °C/s) were run in ViiA 7 real-time PCR system. The resulting melt curves and temperature shift were analyzed by Protein Thermal Shift Software v1.2 (Invitrogen).

### Statistics

Data were presented as the means ± standard deviation (SD) from 3 independent experiments. Comparisons were made by using Student’s *t* test or one-way analysis of variance (ANOVA) according to the data property and experimental design. A significance level was defined as *P* < 0.05.

## Additional Information

**How to cite this article**: Tsang, W. P. *et al*. Icaritin enhances mESC self-renewal through upregulating core pluripotency transcription factors mediated by ERα. *Sci. Rep.* **7**, 40894; doi: 10.1038/srep40894 (2017).

**Publisher's note:** Springer Nature remains neutral with regard to jurisdictional claims in published maps and institutional affiliations.

## Supplementary Material

Supplementary Information
