# Supplementary material for: Icaritin enhances mESC self-renewal through upregulating core pluripotency transcription factors mediated by ERα
Source: Sci Rep. 2017 Jan 16;7:40894. doi: 10.1038/srep40894 (PMC5238509; doi:10.1038/srep40894)
Supplement: Supplementary Information [file srep40894-s1.pdf]

Supplementary Information

Title of manuscript :

Icaritin enhances mESC self-renewal through upregulating core pluripotency transcription factors mediated by ERα

Authors:

Wing Pui Tsang[1,2], Fengjie Zhang[1,2], Qiling He[3], Waijiao Cai[4], Jianhua Huang[4], Wai Yee Chan[1,2], Ziyin Shen[4], Chao Wan[1,2]

Gene	Primer sequences for real-time PCR (5' to 3')
Oct-4	F : GTTTTGGATATGGGTTGAAATATTG
	R: CCACCCTCTAACCTTAACCTCTAAC
Nanog	F : TATGGATTAATTTATTAAGGTAGTT
	R : CTATTCTCCCAAACACCCAAAC
Sox2	F : TATTTTATGTATTTAAGAGAGAGTTAATA
	R : CAACCATCCATATAATAAAAACTATCAAA
Klf-4	F : TTTTATATATATATATTTTTAATTTTTTGT
	R : CTTCTTACTTATAACTTCT
Cyclin A	F : AGTACAGGAGGACCTGTGGC
	R : ATTGACCCCATGGTCAGAGA
Cyclin B	F : ACCAGAGGTGGAACTTGCTG
	R : GGCTTGGAGAGGGATTATCA
Cyclin D1	F : TCCTCTCCAAAATGCCAGAG
	R : GGGTGGGTTGGAAATGAAC
Cyclin E	F : TTGCAAGACCCAGATGAAGA
	R : TCCACGCATGCTGAATTATC
p21	F : CGGTGTCAGAGTCTAGGGGA
	R : ATCACCAGGATTGGACATGG
p27	F : AGTGTCCAGGGATGAGGAAG
	R : GGGGAACCGTCTGAAACATT
p57	F : CTGAAGGACCAGCCTCTCTC
	R : TTCTCCTGCGCAGTTCTCTT
beta-Actin	F : CCCAGAGCAAGAGAGG
	R : GTCCAGACGCAGGATG

Supplementary Table S1. List of primers sequences for quantitative real-time PCR.

Symbol	Gene name	Fold change (Icaritin/Control)	P-value	Up/Down regulation
Acvr1	Activin A receptor 1	1.69	0.002	Up
Acvr1b	Activin A receptor 1B	1.11	0.253	Up
Acvr1c	Activin A receptor IC	**-1.03**	0.106	Down
Acvr2a	Activin receptor IIA	1.05	0.196	Up
Acvr2b	Activin receptor IIB	1.13	0.079	Up
Acvrl1	Activin A receptor-like 1	**-1.15**	0.014	Down
Amhr2	Anti-Mullerian hormone type 2 receptor	1.33	0.000	Up
Bcl9	B-cell CLL/lymphoma 9	1.08	0.286	Up
Bcl9l	B-cell CLL/lymphoma 9-like	1.07	0.447	Up
Bmpr1a	Bone morphogenetic protein receptor 1A	1.07	0.012	Up
Bmpr1b	Bone morphogenetic protein receptor 1B	1.18	0.154	Up
Bmpr2	Bone morphogenic protein receptor II	1.21	0.003	Up
Cdx2	Caudal type homeobox 2	**-1.33**	0.097	Down
Crebbp	CREB binding protein	1.33	0.004	Up
Ctnnb1	Catenin beta 1	1.15	0.010	Up
E2f5	E2F transcription factor 5	**-1.07**	0.026	Down
Eng	Endoglin	1.06	0.134	Up
Ep300	E1A binding protein p300	1.08	0.175	Up
Fgfr1	Fibroblast growth factor receptor 1	**-1.04**	0.116	Down
Fgfr2	Fibroblast growth factor receptor 2	1.17	0.024	Up
Fgfr3	Fibroblast growth factor receptor 3	1.31	0.414	Up
Fgfr4	Fibroblast growth factor receptor 4	1.10	0.197	Up
Fzd1	Frizzled homolog 1 (Drosophila)	1.13	0.138	Up
Fzd2	Frizzled homolog 2 (Drosophila)	1.26	0.112	Up
Fzd3	Frizzled homolog 3 (Drosophila)	2.82	0.000	Up
Fzd4	Frizzled homolog 4 (Drosophila)	1.10	0.078	Up
Fzd5	Frizzled homolog 5 (Drosophila)	1.04	0.308	Up
Fzd6	Frizzled homolog 6 (Drosophila)	1.03	0.286	Up
Fzd7	Frizzled homolog 7 (Drosophila)	1.13	0.050	Up
Fzd8	Frizzled homolog 8 (Drosophila)	1.25	0.001	Up
Fzd9	Frizzled homolog 9 (Drosophila)	1.00	0.939	---
Gli1	GLI-Kruppel family member GLI1	1.08	0.395	Up
Gli2	GLI-Kruppel family member GLI2	**-1.14**	0.301	Down
Gli3	GLI-Kruppel family member GLI3	**-1.08**	0.138	Down
Il6st	Interleukin 6 signal transducer	1.01	0.592	Up
Lef1	Lymphoid enhancer binding factor 1	1.46	0.000	Up
Lifr	Leukemia inhibitory factor receptor	**-1.11**	0.010	Down
Lrp5	Low density lipoprotein receptor-related protein 5	**-1.02**	0.506	Down
Lrp6	Low density lipoprotein receptor-related protein 6	1.04	0.319	Up
Ltbp1	Latent transforming growth factor beta binding protein 1	**-1.08**	0.122	Down
Ltbp2	Latent transforming growth factor beta binding protein 2	**-1.14**	0.039	Down
Ltbp3	Latent transforming growth factor beta binding protein 3	1.14	0.065	Up
Ltbp4	Latent transforming growth factor beta binding protein 4	1.41	0.009	Up
Ncstn	Nicastrin	1.24	0.002	Up
Nfat5	Nuclear factor of activated T-cells 5	1.27	0.063	Up
Nfatc1	Nuclear factor of activated T-cells 1	**-1.04**	0.543	Down
Nfatc2	Nuclear factor of activated T-cells 2	**-1.18**	0.081	Down
Nfatc3	Nuclear factor of activated T-cells 3	1.11	0.059	Up
Nfatc4	Nuclear factor of activated T-cells 4	**-1.19**	0.003	Down
Notch1	Notch gene homolog 1 (Drosophila)	1.01	0.922	Up
Notch2	Notch gene homolog 2 (Drosophila)	**-1.09**	0.035	Down
Notch3	Notch gene homolog 3 (Drosophila)	1.04	0.368	Up
Notch4	Notch gene homolog 4 (Drosophila)	1.51	0.001	Up
Psen1	Presenilin 1	1.26	0.005	Up
Psen2	Presenilin 2	**-1.10**	0.100	Down
Psenen	Presenilin enhancer 2 homolog (C. elegans)	1.11	0.199	Up
Ptch1	Patched homolog 1	1.08	0.242	Up
Ptchd2	Patched domain containing 2	1.00	0.757	---
Pygo2	Pygopus 2	1.02	0.741	Up
Rbl1	Retinoblastoma-like 1 (p107)	1.09	0.214	Up
Rbl2	Retinoblastoma-like 2	**-1.41**	0.005	Down
Rbpjl	Recombination signal binding protein for immunoglobulin kappa J region-like	1.96	0.314	Up
Rgma	RGM domain family, member A	**-1.28**	0.001	Down
Smad1	MAD homolog 1 (Drosophila)	1.28	0.006	Up
Smad2	MAD homolog 2 (Drosophila)	1.01	0.746	Up
Smad3	MAD homolog 3 (Drosophila)	1.01	0.768	Up
Smad4	MAD homolog 4 (Drosophila)	1.00	0.946	---
Smad5	MAD homolog 5 (Drosophila)	**-1.04**	0.316	Down
Smad6	MAD homolog 6 (Drosophila)	1.21	0.026	Up
Smad7	MAD homolog 7 (Drosophila)	1.05	0.273	Up
Smad9	MAD homolog 9 (Drosophila)	**-1.37**	0.001	Down
Smo	Smoothened homolog (Drosophila)	1.16	0.673	Up
Sp1	Trans-acting transcription factor 1	1.43	0.001	Up
Stat3	Signal transducer and activator of transcription 3	1.06	0.290	Up
Sufu	Suppressor of fused homolog (Drosophila)	1.18	0.021	Up
Tcf7	Transcription factor 7, T-cell specific	1.06	0.124	Up
Tcf7l1	Transcription factor 7-like 1 (T-cell specific, HMG box)	1.35	0.001	Up
Tcf7l2	Transcription factor 7-like 2, T-cell specific, HMG-box	**-1.03**	0.516	Down
Tgfbr1	Transforming growth factor, beta receptor I	**-1.02**	0.610	Down
Tgfbr2	Transforming growth factor, beta receptor II	**-1.24**	0.020	Down
Tgfbr3	Transforming growth factor, beta receptor III	1.29	0.001	Up
Tgfbrap1	Transforming growth factor, beta receptor associated protein 1	1.32	0.012	Up
Vangl2	Vang-like 2 (van gogh, Drosophila)	1.26	0.000	Up
Zeb2	Zinc finger E-box binding homeobox 2	**-1.01**	0.738	Down

Supplementary Table S2. A summary of gene names, fold-change of transcript expression relative to the control cells, and *P* values were shown.

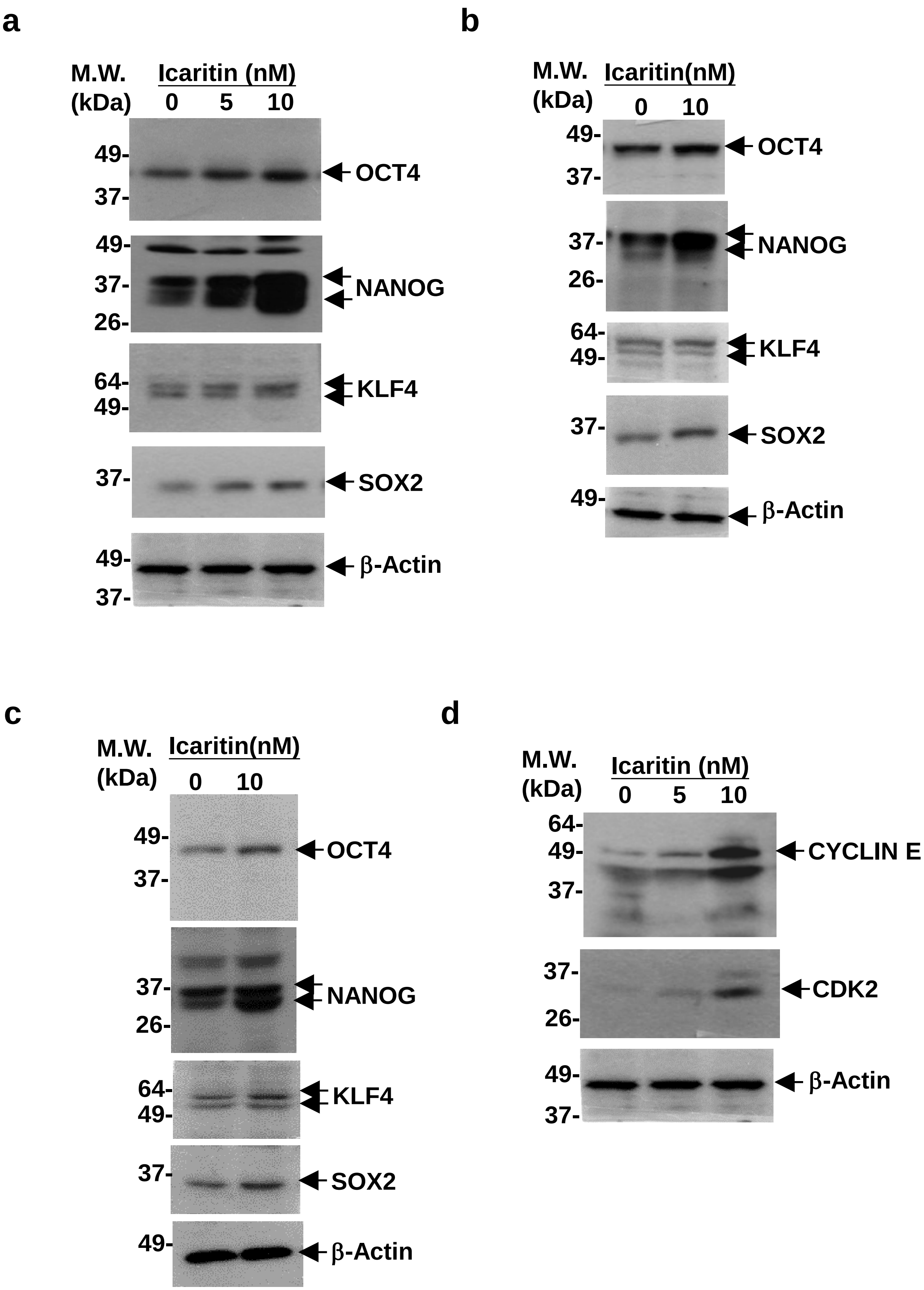
a
M.W. (kDa)
Icaritin (nM)
0 5 10
49-
37-
OCT4
49-
37-
26-
NANOG
64-
49-
KLF4
37-
SOX2
49-
37-
β-Actin
b
M.W. (kDa)
Icaritin(nM)
0 10
49-
37-
OCT4
37-
26-
NANOG
64-
49-
KLF4
37-
SOX2
49-
β-Actin
c
M.W. (kDa)
Icaritin(nM)
0 10
49-
37-
OCT4
37-
26-
NANOG
64-
49-
KLF4
37-
SOX2
49-
β-Actin
d
M.W. (kDa)
Icaritin (nM)
0 5 10
64-
49-
37-
CYCLIN E
37-
26-
CDK2
49-
37-
β-Actin

e

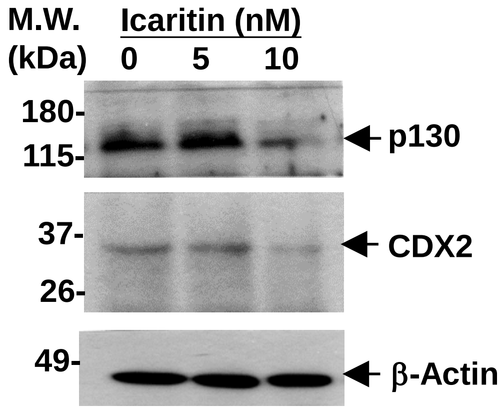
M.W.
(kDa)
Icaritin (nM)
0
5
10
180-
115-
p130
37-
26-
CDX2
49-
β-Actin

f

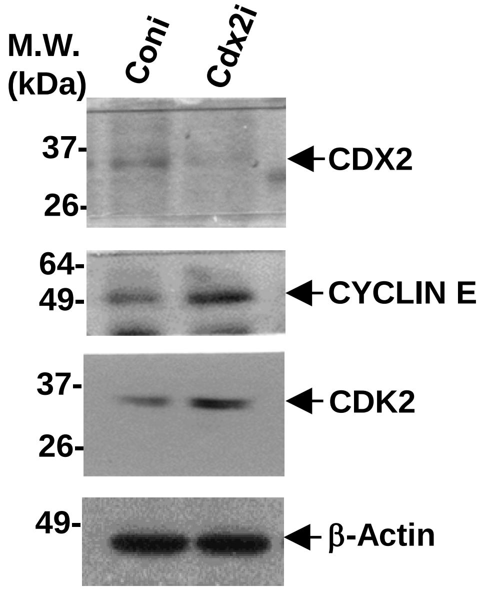
M.W.
(kDa)
Coni
Cdx2i
37-
26-
CDX2
64-
49-
CYCLIN E
37-
26-
CDK2
49-
β-Actin

g

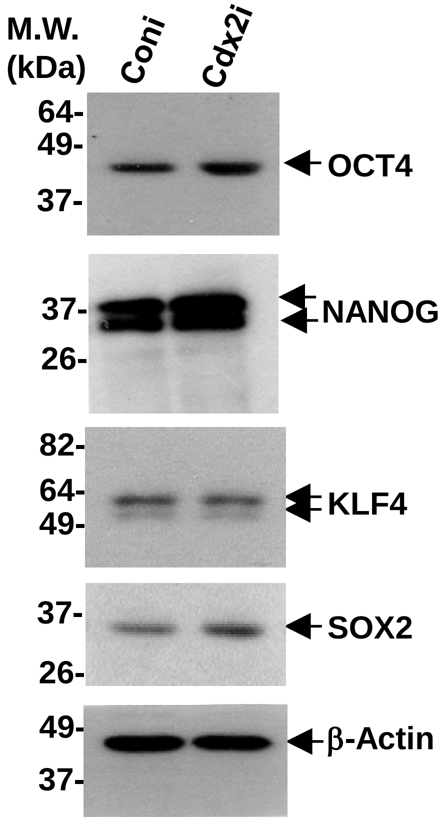
M.W.
(kDa)
Coni
Cdx2i
64-
49-
37-
OCT4
37-
26-
NANOG
82-
64-
49-
KLF4
37-
26-
SOX2
49-
37-
β-Actin

h

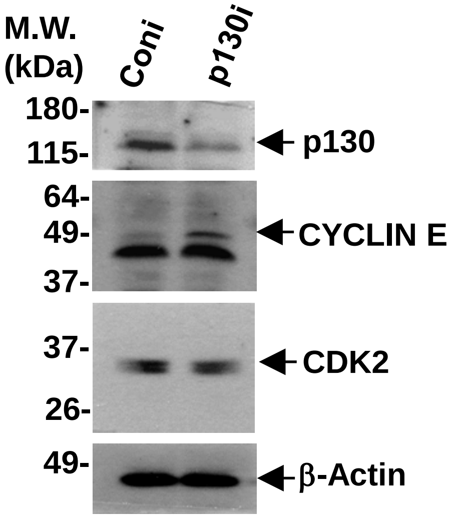
M.W.
(kDa)
Coni
p130i
180-
115-
p130
64-
49-
37-
CYCLIN E
37-
26-
CDK2
49-
β-Actin

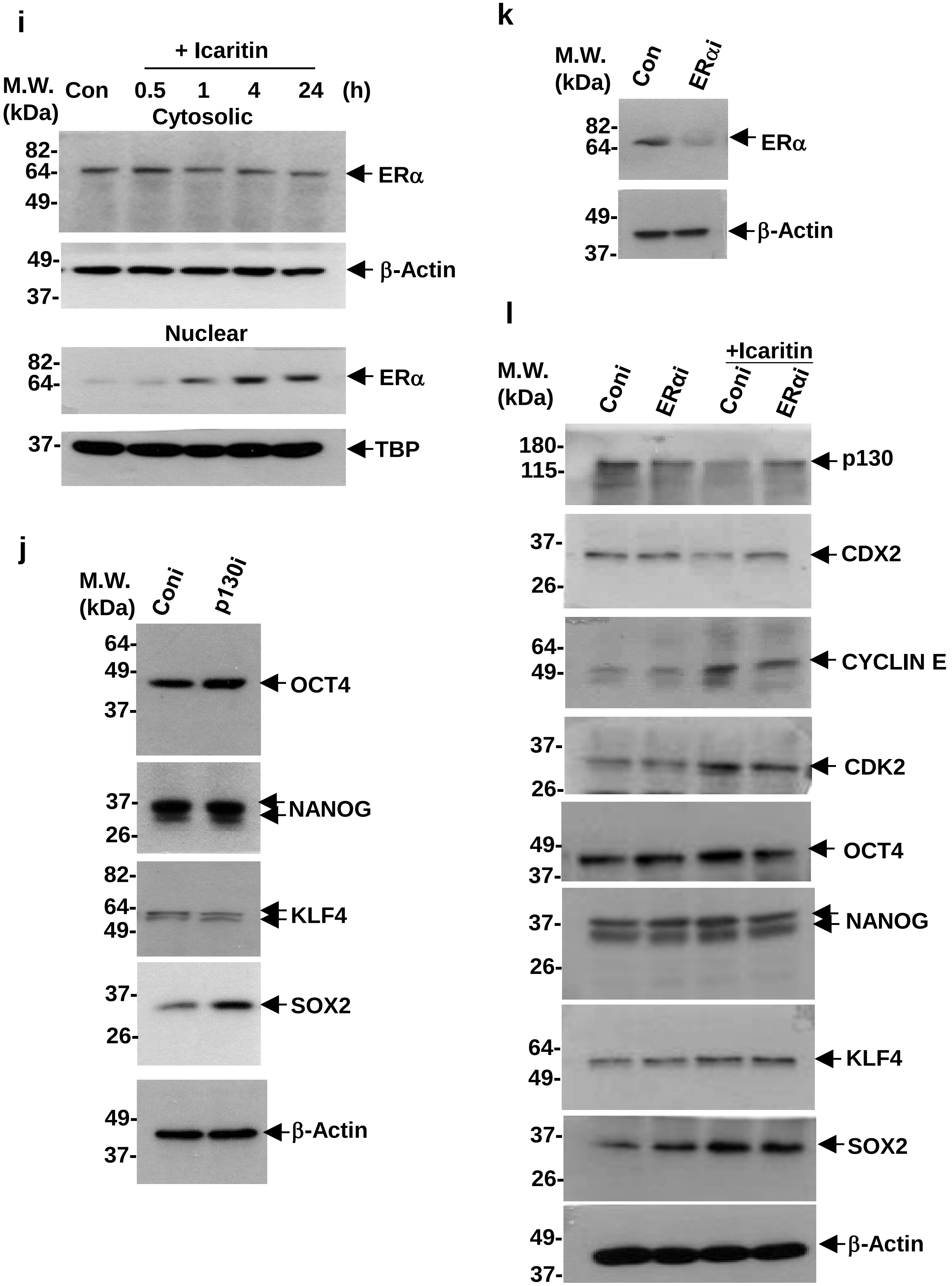
i
+ Icaritin
M.W. (kDa)
Con
0.5
1
4
24
(h)
Cytosolic
82-
64-
49-
ERα
49-
37-
β-Actin
Nuclear
82-
64-
ERα
37-
TBP
j
M.W. (kDa)
Coni
p130i
64-
49-
37-
OCT4
37-
26-
NANOG
82-
64-
49-
KLF4
37-
26-
SOX2
49-
37-
β-Actin
k
M.W. (kDa)
Con
ERαi
82-
64-
ERα
49-
37-
β-Actin
l
+Icaritin
M.W. (kDa)
Coni
ERαi
Coni
ERαi
180-
115-
p130
37-
26-
CDX2
64-
49-
CYCLIN E
37-
26-
CDK2
49-
37-
OCT4
37-
26-
NANOG
64-
49-
KLF4
37-
26-
SOX2
49-
37-
β-Actin

Supplementary Figure S3. Full Western blots of Fig. 1-8.

Fig. S3a: full Western blot of Fig.1f.

Fig. S3b. full Western blot of Fig.2b.

Fig. S3c. full Western blot of Fig.2c.

Fig. S3d. full Western blot of Fig.3c.

Fig. S3e. full Western blot of Fig.4c.

Fig. S3f. full Western blot of Fig.5a.

Fig. S3g. full Western blot of Fig.5b.

Fig. S3h. full Western blot of Fig.6a.

Fig. S3i. full Western blot of Fig.6b.

Fig. Sj. full Western blot of Fig.7d.

Fig.S3k. full Western blot of Fig. 8a.

Fig. S3l. full Western blot of Fig. 8e.
